# Spatiotemporal evolution of epileptic seizure based on mutual information and dynamic brain network

**DOI:** 10.1186/s12911-021-01439-4

**Published:** 2021-07-30

**Authors:** Mengnan Ma, Xiaoyan Wei, Yinlin Cheng, Ziyi Chen, Yi Zhou

**Affiliations:** 1 School of Biomedical Engineering, Sun Yat-sen University, No. 132 Waihuan East Road, Guangzhou, 510006 China; 2 Department of Medical Informatics, Zhongshan School of Medicine, Sun Yat-sen University, No. 74 Zhongshan 2nd Road, Guangzhou, 510080 China; 3 Minister of Science, Education and Data Management Department, Guangzhou Women and Children’s Medical Center, National Children’s Medical Center for South Central Region, Guangzhou Medical University, No. 9 Jinsui Road, Guangzhou, 510623 China; 4 Department of Neurology, First Affiliated Hospital, Sun Yat-sen University, No. 58 Zhongshan 2nd Road, Guangzhou, 510080 China; 5 Key Laboratory of Tropical Disease Control (Sun Yat-sen University), Ministry of Education, No. 74 Zhongshan 2nd Road, Guangzhou, 510080 China

**Keywords:** Epilepsy, Brain Network, Mutual Information, Complex Network

## Abstract

**Background:**

Epilepsy was defined as an abnormal brain network model disease in the latest definition. From a microscopic perspective, it is also particularly important to observe the Mutual Information (MI) of the whole brain network based on different lead positions.

**Methods:**

In this study, we selected EEG data from representative temporal lobe and frontal lobe epilepsy patients. Based on Phase Space Reconstruction and the calculation of MI indicator, we used Complex Network technology to construct a dynamic brain network function model of epilepsy seizure. At the same time, about the analysis of our network, we described the index changes and propagation paths of epilepsy discharge in different periods, and spatially monitors the seizure change process based on the analysis of the parameter characteristics of the complex network.

**Results:**

Our model portrayed the functional synergy between the various regions of the brain and the state transition during the seizure process. We also characterized the EEG synchronous propagation path and core nodes during seizures. The results shown the full node change path and the distribution of important indicators during the seizure process, which makes the state change of the seizure process more clearly.

**Conclusion:**

In this study, we have demonstrated that synchronization-based brain networks change with time and space. The EEG synchronous propagation path and core nodes during epileptic seizures can provide a reference for finding the focus area.

## Background

Deep learning can autonomously learn information from epilepsy EEG signals, and macroscopically classify and give advance warning of different periods seizure progress from the time dimension [1–3]. Moreover, the spatial network connected by the brain leads contains important information. Epilepsy was defined as an abnormal brain network model disease in the latest definition [4–6]. From a microscopic perspective, it is also particularly important to observe MI of the whole brain network based on different lead positions. About the brain network model, the major research points are on the propagation of EEG signals in the brain network model and the information interaction between brain different regions and periods, which is helpful to gain a deeper understanding of the whole process mechanism of the seizure brain network [7].

Brain discharge activity has the characteristics of transmission [8]. There will be some synchronization correlation between different signals. Brain network is a complex network with connections during static and dynamic brain activities. And its connection patterns are closely related to behavioral awareness [9]. EEG can record the different brain regions’time series signals, which can reflect the activity and coordination between the brain regions. Furthermore, the relationship between brain regions can be analyzed through the brain network. The theory of MI can be used to reveal the inherent hidden relationships between synchronization signals [10]. However, most of them are based on the original signal, without considering the phase space reconstruction of the data. On the basis of synchronization, complex network technologies are often used to build brain network models. Yet now, most studies only selected two periods for researching, and did not involve more studies on the transition state of epilepsy in more different periods.

In our study, we selected EEG data from the representative temporal and frontal lobe epilepsy patients based on strict data selection criteria. Based on the phase space reconstruction and the calculation of mutual information indicators, complex network technology was used to construct a dynamic brain network function model of epilepsy seizure. At the same time, the parameter characteristics of the complex network were analyzed to describe the index changes and propagation paths of epilepsy discharge in different periods. And the seizure process is monitored spatially.

## Related work

### Advances in research on brain network of seizures

Deep learning can perform automatic feature extraction for epilepsy EEG and macroscopic examination of the seizure onset period from the time dimension [11, 12]. Moreover, the recognition of pre-ictal is identified to achieve the early seizure warning. In another aspect, the spatial network connected by the brain leads contains important information [2, 13]. From a microscopic perspective, it is also particularly important to observe MI of the whole brain network based on different lead positions. Studies in the past few years shown that The Network Connected Epilepsy Center (the concept of brain network) which means more complex ”epilepsy network”, have already replaced the classic, simple single ”epilepsy” concept [14]. In the concept of ”epilepsy network”, Synchronized activities of ”nodes” with increased excitability (or decreased inhibition) were involved in the occurrence of pathological epilepsy. Any effect on ”nodes” of the epilepsy brain will affect the activity of other parts of the brain through the brain network.

Understanding the interrelationships between different brain regions and different nodes may help us to have a more complete understanding on the ongoing brain information interaction. Furthermore, it helps to explore the mechanism of brain seizures. In view of the EEG acquisition method, the polar lead serves as a basic node to record the information of various parts of the brain. At the same time, the lead transmitted to other brain regions/nodes and affected the activities of other brain regions/nodes [15]. The calculation of the degree of synchronization effect is called synchronization measurement, including linear and nonlinear methods [16]. The linear methods are to calculate the direct correlation of the two variables from the time domain or frequency domain linear methods [17]. Non-linear methods are such as information transfer complexity, non-linear indicators, etc [18]. The calculation of mutual information according to the time window is generally used for short-term synchronization [10]. And the non-linear indicator can support the synchronization calculation of a longer time window [19]. Different measurement formulas captured different signal synchronization situations. The node propagation will have different impacts by different distance. the nodes in the ipsilateral brain area propagate fast and the nodes in the heteromeral brain area propagate slowly. That is the reason of the problem of synchronization delay of network nodes.

On the basis of synchronous indicators, Graph Theory technology is used to construct the functional network topology [20]. A large number of experiments have shown that its network topology changed with the continuous progress of the attack, with regularity in the irregularity. Preliminary researches show that in 70% of patients, their brains are accompanied by a process of synchronous information enhancing and weakening during different seizure periods. Literature [14] showed that the intracranial EEG signals of 8 epileptic patients revealed that the network was moving in a more orderly direction: Such as a higher clustering coefficient during the seizure and a shorter path length. However, the network is random and disordered during the inter-seizure period. Hao [21] showed that the aggregation degree of the core nodes during ictal period was significantly higher than interictal period, but the path length of the information transmission did not change significantly. Literature [22] used directed network outflow density indicator to describe the early characteristics of epileptic areas before seizure. The information flow always came from the same side, and are independent of the onset side area. However, most studies only select two or three periods for researching, and do not involve more studies on the transition state of epilepsy in more different periods [14, 23–25]. To sum up, although there are so many studies on epilepsy monitor, it has not yet entered clinical application. The problem of epilepsy monitor is attributed to the study of spatiotemporal monitoring in different periods. And the key to accurate identification is to determine the pre-onset stage in combination with nonlinear dynamic changes. Traditional seizure detection and prediction are mostly based on small sample machine learning methods [26]. There is no pre-research on long-term large-scale data. Based on the diversity of clinical epilepsy types and actual needs, our study will select specific epilepsy patients’ data to carry out systematic long-term seizure monitoring research and synchronize brain network function changes in different periods. We hope to promote the researches on seizure prediction and detection tasks and brain network Changes.

### Complex network and brain network

#### Brain network

In the real world, there are a variety of auxiliary network applications, such as social network, protein network, interpersonal network and transportation networks [7]. Complex Network method is an important tool for constructing a network, measuring network indicators, and understanding the information transmission paths in the network [27, 28]. The essence of Complex Networks comes from a branch of mathematics – Graph Theory. Complex Network forms a network model by reducing them to a set of nodes and abstract connections [29]. It provides an excellent tool for studying all aspects of the brain network. The Complex Network was described mathematically based on graph theory technology, and was estimated by probability theory and statistics and dynamic system theory.

Brain network is an important embodiment of the application of complex network technology in brain studies [30]. The nodes are defined as EEG channels, and the edge is a direct embodiment of the elements of the node association matrix. Graph Theory can be used to identify key nodes and subnetworks. Researches on brain network can be divided into structural brain network and functional brain network [31]. Structural brain network placed emphasis on neuroanatomy. Functional network focused on the interaction of EEG signals. In our research, we mainly focus on the construction of functional network.

#### Parameter features of complex networks

The complex network of brain built by EEG data pay close attention to brain network activity indicators and information interaction at different periods [32]. In the complex network model, there are corresponding characteristics measurement indicators for different network nodes and edges. The most basic and commonly used indicators are clustering coefficient, path length, degree and degree distribution

1. clustering coefficient and average clustering coefficient Clustering coefficient is often used to measure part of the connectivity within the network. It can be expressed as the aggregation level of a certain node and its neighbors. As a general rule, we look at the entire model level globally. That’s why we can use average clustering coefficient as a measurement indicator to represent the measurement of global connectivity of the entire graph.
2. average path length The path describes how fast the information flows. Path length refers to the number of steps that a network node to reach another node. Intuitively, in the network, when the average path length is short, it is easy to go from one point to another random point. The average path length is mainly used to determine whether the network model exhibits small-world network attributes.
3. degree and degree distribution The degree of nodes and the distribution of nodes can reflect the relative importance of the nodes, intuitively, the number of edges connected between a certain node and other nodes. In our study, the above three indicators were selected as indicators to measure network synchronization.

## Materials and methods

### Data

#### Data selection criteria

In order to ensure the construction of the brain network and avoid the generation of artifacts as much as possible, our data were selected in strict accordance with the following criteria:

1. Must be patients with focal epilepsy;
2. Have obvious seizures and EEG signals;
3. Each patient’s long-term EEG must include at least 2 or more seizures.

#### Data details and preprocessing

Based on our data selection criteria, we selected two epilepsy patients. They are patients with temporal lobe epilepsy (Temporal Lobe Epilepsy, TLE) and patients with frontal lobe epilepsy (Frontal Lobe Epilepsy, FLE). And We used their data to build a brain network model for comparison. The specific information of the data is shown in Table 1. The calculation data mainly adopted the entire process of each seizure selection including inter-seizure period, pre-seizure period, and seizure period. At the same time, it was compared with the EEG signals during sleep. Each episode was divided into 2 to 6 segments, each segment is 20s long. And the same length data segments were used to calculate the degree of synchronization.

**Table 1:** Experimental data specific information

Patient	Gender	Ages	Leads	State	Monitor time	Seizures	Seizure time
TLE	Female	28	22	W$\rightarrow$SP	24H	5	824s
FLE	Male	39	22	W$\rightarrow$SP	24H	4	895s

### Methods

#### Research framework

**Fig. 1:**
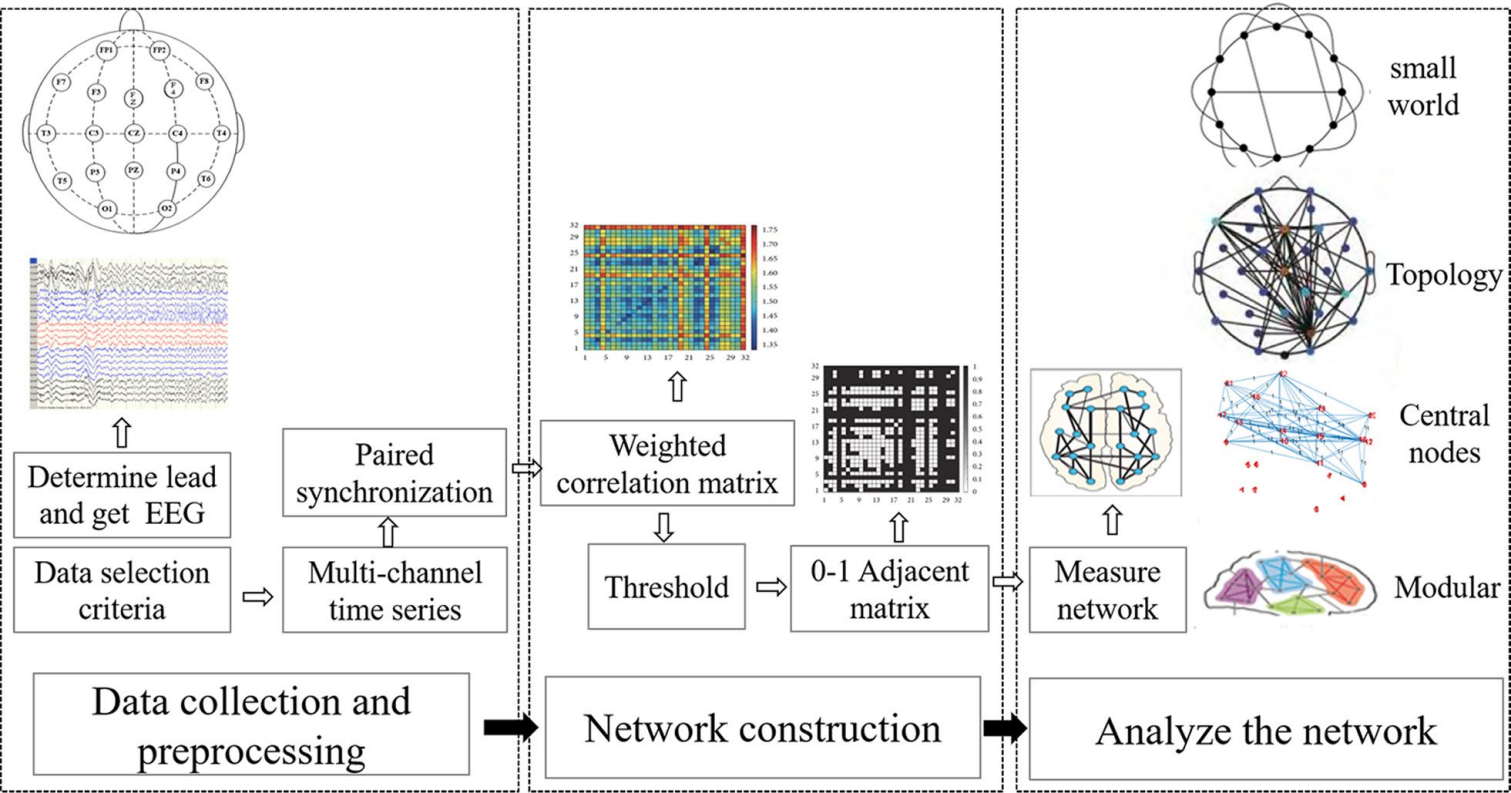
Research framework of brain network system

Figure 1 shows the overall framework of this study. First, we selected the EEG data of representative temporal lobe and frontal lobe epilepsy patients according to the data selection criteria. For the multi-channel time series EEG data, we calculated the synchronization index based on Phase Space Reconstruction and MI. After that, we used the uniform MI of all channels as the threshold and used complex network technology to build a dynamic brain network function model of seizure. Finally, the analysis of our network was based on the analysis of parameter characteristics of complex network. Our model described the index changes and propagation paths of epilepsy discharge in different periods. We spatially monitor the evolution of seizure.

#### Synchronization calculation

There are four kinds of synchronization measures [33–35]: such as correlation calculation, phase calculation, information-based synchronization index and Granger causality measurement. In this article, we adopted the most commonly method of mutual information. Here, the original EEG data was not used to directly obtain the mutual information. Considering that neuroelectric signals are two-dimensional displays of nonlinear systems, we used the multi-dimensional nonlinear system reconstructed by phase space to perform the calculation. The mathematical principle is as following:

For the X leads’collected EEG signals, a scatter plot is drawn between every two leads. And to obtain the maximum value of mutual information in different regions gridded every two leads. We set the collected EEG signal $X_{k,i}$ to M channels $(k=1,\cdots ,M)$, *N* time points $(i=1,\cdots ,N)$, and use phase space to reconstruct an embedded vector $X_{k,i}=(x_{k,i},x_{k,i+l},x_{k,i+2l},\cdots ,x_{k,i+\left( m-1\right) l})$, Where *l* is the amount of delay and *m* is the number of dimensions.

The vector set can be expressed as:

1 $$\begin{aligned} \left[ \begin{array}{cccc} x_1 &{} x_2 &{} \cdots &{} x_m \\ &{} &{} \ddots &{} \vdots \\ x_{n-m+1} &{} x_{n-m+2} &{} \cdots &{} x_n \\ \end{array}\right] \end{aligned}$$

These vectors have a certain probability of being scattered in the phase space. The result of lead a by phase space to reconstructed is $\left[ s_1,s_2,\cdots ,s_n\right]$, The corresponding probability is scattered as $\left[ p_s\left( s_1\right) ,p_s\left( s_2\right) ,\cdots ,p_s\left( s_n\right) \right]$; The result of lead b by phase space to reconstructed is $\left[ q_1,q_2,\cdots ,q_n\right]$, The corresponding probability is scattered as $\left[ p_q\left( q_1\right) ,p_q\left( q_2\right) ,\cdots ,p_q(q_n)\right]$. The information entropy of lead a and lead b can be obtained from Shannon entropy formula:

2 $$\begin{aligned} H(S)= & {} -\sum _{i}^{}p_s(s_i)\log _2p_s(s_i) \end{aligned}$$

3 $$\begin{aligned} H(Q)= & {} -\sum _{j}^{}p_q(q_j)\log _2p_q(q_j) \end{aligned}$$

Then the joint entropy of the two leads can be obtained from The joint Shannon entropy formula:

4 $$\begin{aligned} H(S,Q)= & {} -\sum _{j,i} p_{qs}(q_j,s_i)log_2p_{qs}(q_j,s_i) \end{aligned}$$

In the end, bring the previous three formulas into Shannon’s formula to get the amount of information between the two channels:

5 $$\begin{aligned} MI(S,Q)= & {} H(S)+H(Q)-H(S,Q) \end{aligned}$$

#### Brain network model

*Construction of brain network*

First, the phase space reconstruction of nonlinear dynamics was used to recalculate the original EEG signal to a multi-dimensional nonlinear system. Then we calculated the mutual information between the leads based on the mutual information entropy to obtain the lead synchronization indicator. After that, we binarized the indicator to reduce the influence of weakly correlated leads (There will be a certain correlation between leads). Whether two nodes have edges depended on the size of the synchronization between the two channels. When the amount of MI is greater than the threshold, there is a connected edge, otherwise no. In this way we get 0-1 binary unauthorized network. If the relationship strength between nodes with connected edges is given to the connected edge weight, in that way, we got weighted network. There are many ways to choose the threshold without a standard conclusion. In this study, the uniform mutual information of all channels was selected as the threshold to complete the construction of the network model.

*Statistical Analysis* We use complex network measurement indicators to analyze the brain network and to analyze the characteristic changes in different seizure periods. At the same time, statistical testing was performed. If the data has a normal distribution and homogeneous variance, then an analysis of variance (ANOVA) model was used for statistics to test different eigenvalues for synchronization; otherwise, a non-parametric test was used. The analysis was performed using SPSS software (version 18) and the inspection level was set to 0.05.

## Results and discussion

### Comparison of different periods under the synchronization index

For patient with temporal lobe, Table 2 shown the statistical characteristics of mutual information indicator. Patient with TLE had the highest EEG mutual information during ictal period. It meant that the network had the highest synchronization during this period. Moreover, the EEG of conscious period had the lowest synchronization. The mutual information between the sleep period and the interictal period was relatively uniform, and the synchronization during the preictal period was slightly lower than ictal period. From the perspective of difference coefficient of variation, the difference of ictal seizure was large, but the awake period was small.

For patient with frontal lobe epilepsy, the mutual information indicators in Table 2 shown the highest EEG synchronization during ictal, followed by pre-ictal. The mutual information indexes of sleep period, interictal period and awake period were relatively uniform. From the coefficient of variation, it was found that the seizure period is the most unbalanced and the sober period was the most consistent. Table 3 shown the statistical test of mutual information indicators in different periods. the ictal, preictal and interictal were less than 0.5, which meant that the mutual information entropy value can distinguish the main indexes of interictal period, preictal period and onset period.

**Table 2:** The synchronization index of different periods

EEG	Average MI		Coefficient of Variation	
	TLE	FLE	TLE	FLE
AWAKE	0.926±0.132	1.007±0.066	7.549±2.619	7.960±1.647
SLEEP	1.062±0.062	1.000±0.143	9.201±2.273	9.472±2.108
ICTAL	1.072±0.119	1.071±0.176	8.431±2.206	10.283±1.744
INTERICTAL	1.062±0.062	1.010±0.143	10.201±2.273	9.472±2.002
PREICTAL	1.032±0.119	1.021±0.176	7.980±1.045	8.534±0.653

**Table 3:** Statistical testing of mutual information indicators in different periods

EEG	Average MI		Coefficient of Variation	
	TLE	FLE	TLE	FLE
SP vs AW	P$<0.05*$	P$>0.05$	P$<0.05*$	P$<0.05*$
SP vs PRE	P$<0.05*$	P$<0.05*$	P$>0.05$	P$<0.05*$
PRE vs ICTAL	P$<0.05*$	P$<0.05*$	P$<0.05*$	P$<0.05*$
INTER vs ICTAL	P$<0.05*$	P$<0.05*$	P$>0.05$	P$<0.05*$
PRE vs INTER	P$>0.05$	P$<0.05*$	P$<0.05*$	P$<0.05*$
K-W test	P$<0.05*$	P$<0.05*$	−	P$<0.05*$

Asterisk means that there is a relationship between the two in the table

### Pathway of synchronous discharge

**Fig. 2:**
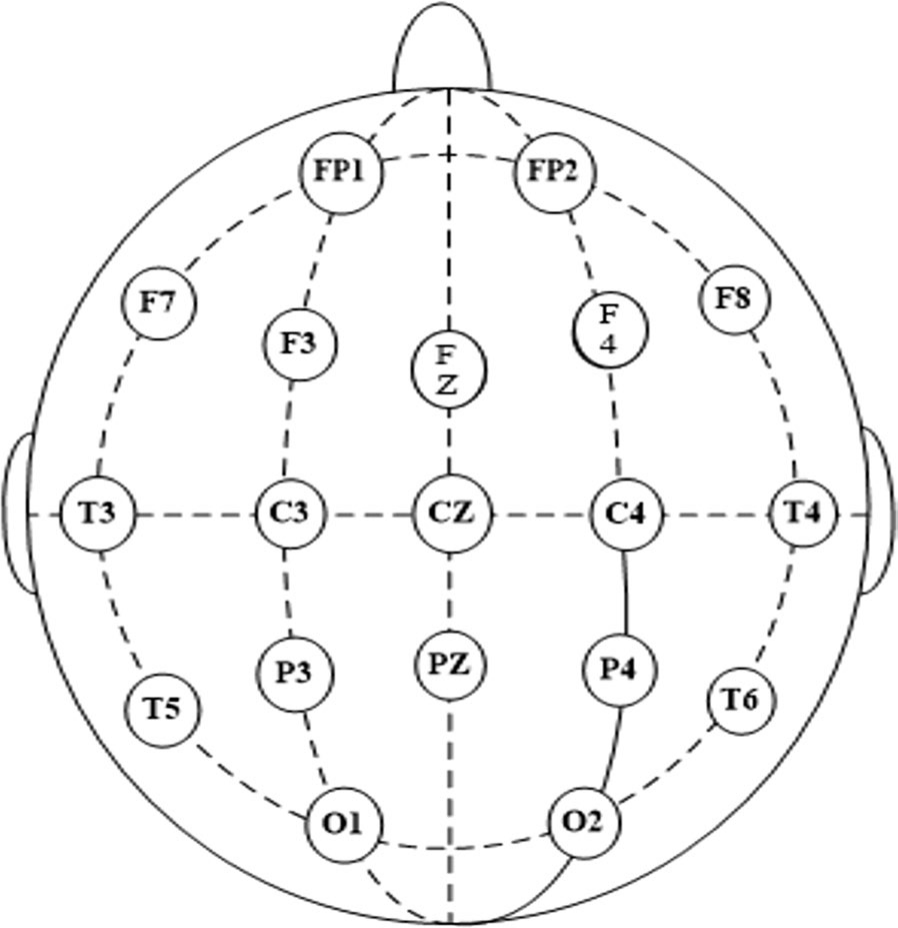
10/20 system electrode placement

Our brain network only shown 20 pairs of channels with the largest mutual information. In order to facilitate calculation, the number sequence was used in Table 4 instead of the corresponding lead name. Electrode placement as shown in Fig. 2. Apart from the 19 identified leads in the figure, it also included reference electrodes m1 and m2, and an additional electrode Afz.

**Table 4:** The corresponding Lead names

Number	Leads	Number	Leads
1	m1	12	Cz
2	F7	13	Pz
3	T3	14	Fp2
4	T5	15	F4
5	Fp1	16	C4
6	F3	17	P4
7	C3	18	O2
8	P3	19	m2
9	O1	20	F8
10	Afz	21	T4
11	Fz	22	T6

**Fig. 3:**
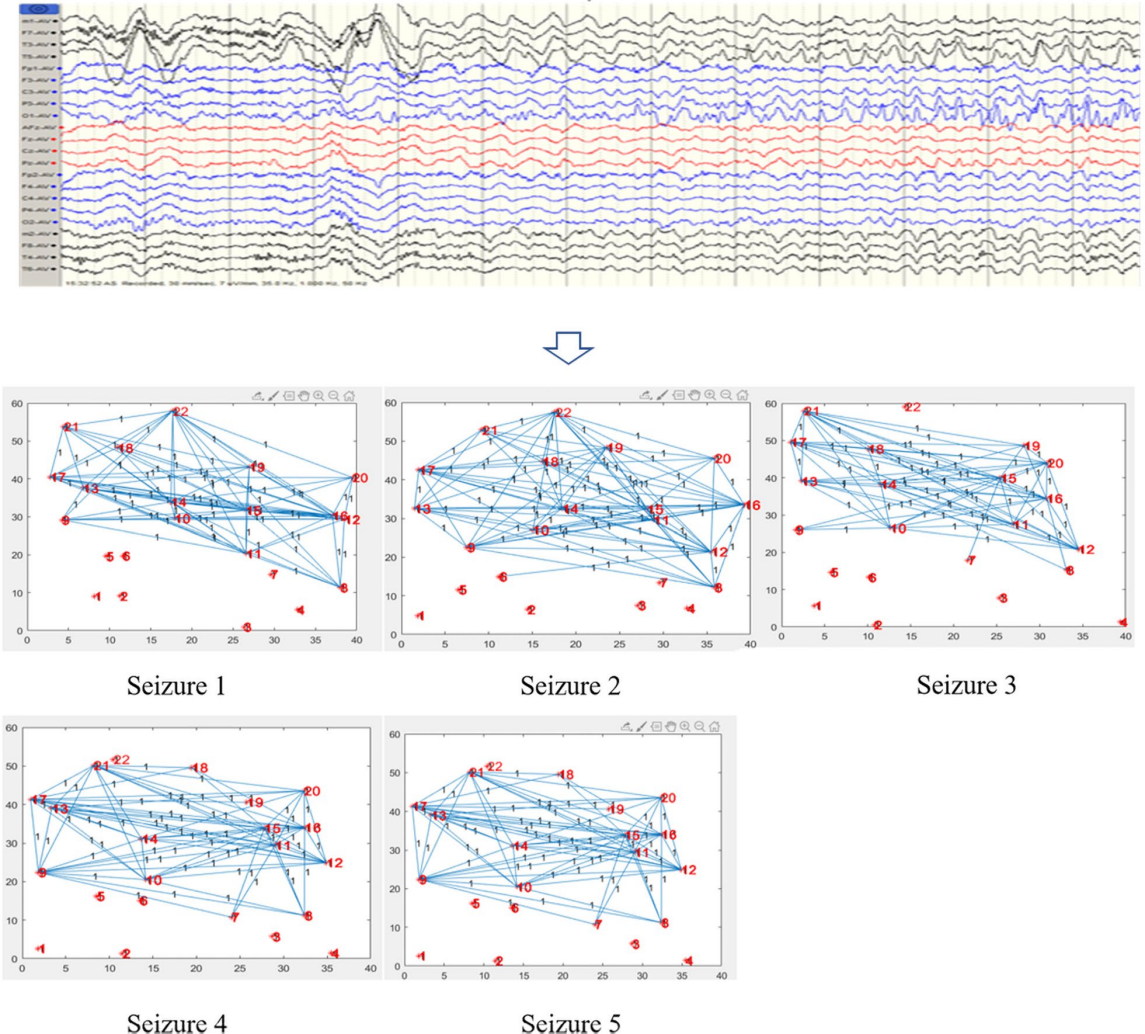
Changes of brain network during seizure in temporal lobe epilepsy

Figure 3 shown that the changes of brain network in the whole process seizure in patients with TLE. The seizure process was divided into 5 periods, each period shown a 20s brain network. The red nodes in the figure were marked with number, and the numbers corresponded to the leads. The connection between node and node meant a propagation relationship. The vertical axis and the horizontal axis represented the range of data connection, which has no practical significance here. Our focus was on the network model. The network shown that the seizure period started from the left temple. Epilepsy seizure started from the left temporal region of the left temple. And continuous spikes can be seen through the left middle temporal region (T3) and the left posterior temporal region (T3 and T5) of the left middle temple and the back temple. During ictal, the network was first activated in the left hindbrain area, then was symmetrically active throughout the brain, and finally remained active in the right forebrain area. This was similar to the clinical diagnosis. The main network path of patient with temporal lobe epilepsy: $(F3-F4-F8)-(C3-O2)-P3-(T3-P3)-F7-T5$.

**Fig. 4:**
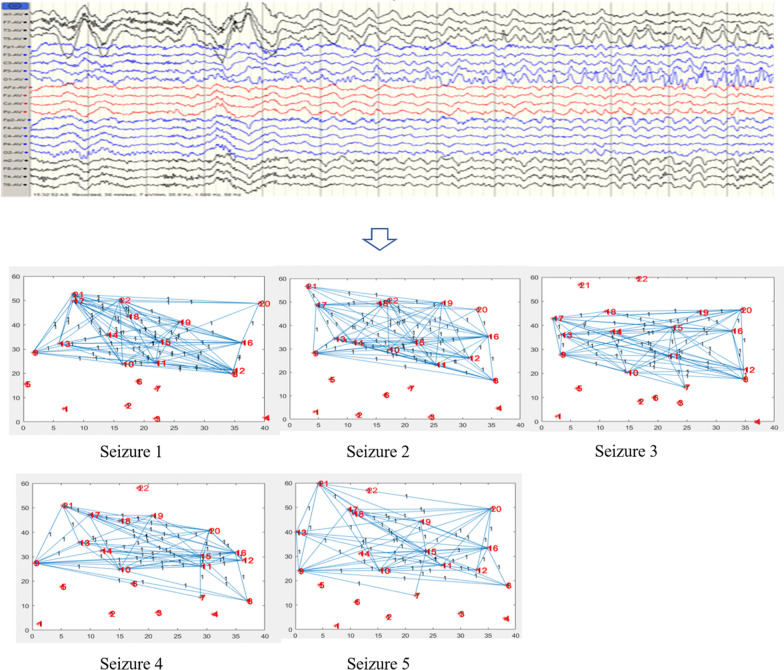
Changes of brain network during seizure in frontal lobe epilepsy

Figure 4 shown that the changes of brain network in the whole process seizure in patients with FLE based on mutual information entropy. The seizure process was divided into 5 periods, each period shown a 20s brain network. The red nodes in the figure were marked with number, and the numbers corresponded to the leads. The connection between node and node meant a propagation relationship. The vertical axis and the horizontal axis represented the range of data connection, which has no practical significance here. Our focus was on the network model. The complex activities of spike and slow wave during the seizure period were mainly in the right anterior zone (F4 and Fp2), and occasionally in the left anterior zone (F3 and Fp1). This involved the entire channels. Some seizure shifted to the anterior-dominated slow activity, involving the right anterior temporal region (F8) and occipital region (O2). Using the network diagram, we can conclude that the network in the prefrontal area during seizure was active. Moreover, the left and right frontal areas were alternately active, followed by the right posterior occipital area. The main network path of patient with frontal lobe epilepsy: $(P4\setminus O1\setminus T4\setminus T5)-(F7\setminus T3)-F4-F7-Fz-(Fp1\setminus Fp2\setminus F4)-(Fp1\setminus F3)$.

### Core nodes of brain network

**Fig. 5:**
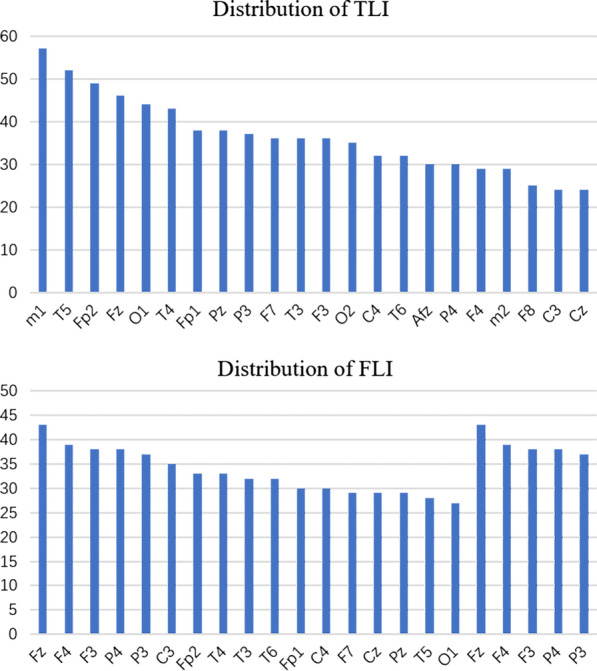
Distribution of core nodes in epilepsy leads

As mentioned before, the construction of the brain network mainly depended on whether a numerical relationship was established between the leads. When there is a relationship, we defined that as 1, otherwise 0. The degree was defined as the number of each lead received from other leads. Degree evaluated the activity of the nodes or leads. We calculated the total number of degrees obtained during the five different periods of the entire episode, and the results are shown in Fig. 5. The lead with the highest node degree is the most active during the entire episode. Combined with the patients’clinical diagnosis reports, we found that patient with temporal lobe epilepsy were more active in T5, O1, T4, F3, and F7, and patient with frontal lobe epilepsy were more active in F4, F3, C4, and C3. The degree distributions of the patients’ different nodes were very close to the clinical neuron discharge frequent area.

### Brain network topology indexes

**Fig. 6:**
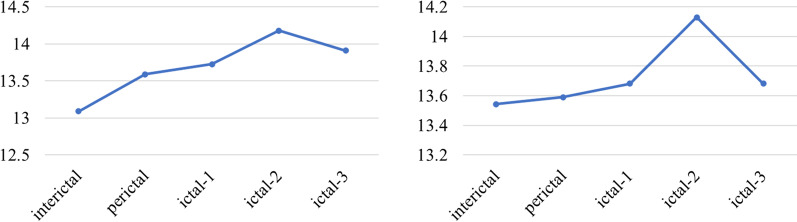
The degree distribution of the whole process of seizure based on the brain network model: Picture on the left was temporal whole brain average degree distribution, picture on the right was frontal lobe whole brain average degree distribution

Here, Fig. 6 shown that the average degree distribution of the two patients were calculated from the inter-seizure period to the post-seizure period. When the interictal transitioned to the ictal, the degree distribution of the entire neural network shown an initial downward trend. During the epileptic seizure, the low-level degree distribution of the brain network shown a slight increase, and then decline. From the analysis of the degree of network information interaction, information interaction was related to degree distribution. The more edges the nodes connected to, the more active the information interaction were. As time progressed, the seizure also followed.

**Fig. 7:**
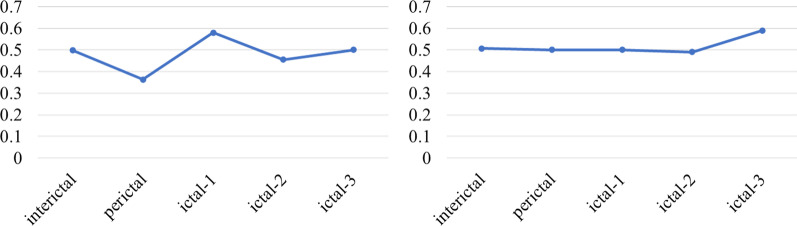
The average path distribution of the whole process of seizures based on brain network model: Picture on the left was temporal whole brain average path distribution, picture on the right was frontal lobe whole brain average path distribution

As can be seen from Fig. 7, patient with temporal lobe and patient with frontal lobe have a stable information transmission path during interictal. In the early stage of seizure, due to the attack was about to arrive, with the excess abnormal discharge, the average path of propagation became shorter. Due to the spread of the brain discharge, the average path to the ictal reached the minimum. Later in the seizure, the seizure gradually ended, the brain resumed normal discharge, and its average path gradually increased.

**Fig. 8:**
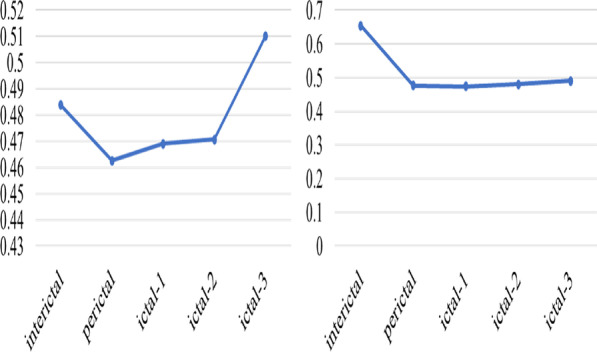
Distribution of average clustering coefficient in the whole process of seizures based on brain network model: Picture on the left was temporal whole brain average clustering coefficient, picture on the right was frontal lobe whole brain average clustering coefficient

For the clustering coefficient, Fig. 8 described the degree of aggregation of the entire network. The higher the value meant the higher the concentration of network nodes. From the beginning of the interictal, the network was highly aggregated and modularized. Some times before ictal period, the clustering coefficient gradually decreased. Considering that excessive brain discharge caused a more fragmented network, the aggregation coefficient decreased. The brain network showed a decentralized trend during the Entire seizure process. Until later stage of the attack, the brain discharge returned to normal and resumed aggregation.

The above network degree distribution and average clustering coefficient shown that both patients with temporal lobe and frontal lobe had similar trends during the state transition. In Clinical practice, from interictal to the middle and late period of seizure, the nerve electrical activity would have a process from continuing to strengthen to stable. This was consistent with changes in the distribution of brain network features. For the path length of discharge propagation, in theory, due to the focal attack, its path distribution fluctuates was less, which was also verified from the average path distribution of patients with the temporal lobe and frontal lobe.

## Conclusions

In our study, we selected EEG data from representative temporal lobe and frontal lobe epilepsy patients. Based on Phase Space Reconstruction and the calculation of MI indicator, we used Complex Network technology to construct a dynamic brain network function model of epilepsy seizure. At the same time, about the analysis of our network, we described the index changes and propagation paths of epilepsy discharge in different periods, and spatially monitors the seizure change process based on the analysis of the parameter characteristics of the complex network.

Judge from the results, we have demonstrated that brain network based on synchronization changed with time and space. EEG synchronous propagation path and core nodes during epileptic seizure can provide a reference for searching for the focal area. Especially mutual information indicators provided quantitative information on the degree of information interaction, which can be consistent with clinical manifestations. EEG signals reflected the discharge of neurons in the brain. The degree of synchronization of EEG signals between the channels represented the strength of information exchange. Therefore, our study can be used as one of the methods to explore the changes of brain network in patients with epilepsy.

## Abbreviations

EEG: Electroencephalogram
MI: Mutual information
TLE: Temporal lobe epilepsy
FLE: Frontal lobe epilepsy
ANOVA: Analysis of variance
SPSS: Statistical product and service solutions
W: Wake
SP: Sleep

## Availability of data and materials

All data were collected from the patients who had been admitted to the hospital, Because of the hospital’s regulations, the data cannot be made publicly available.

## References

[1] Truong ND, Nguyen AD, Kuhlmann L, Bonyadi MR, Yang JW, Ippolito S, Kavehei O (2018). Convolutional neural networks for seizure prediction using intracranial and scalp electroencephalogram. Neural Netw..

[2] Wei XY, Zhou L, Zhang Z, Chen ZY, Zhou Y (2019). Early prediction of epileptic seizures using a long-term recurrent convolutional network. J Neurosci Methods.

[3] Yao X, Cheng Q, Zhang G-Q: A novel independent rnn approach to classification of seizures against non-seizures. arXiv preprint arXiv:1903.09326 (2019)

[4] Omidvarnia A, Kowalczyk MA, Pedersen M, Jackson GD (2019). Towards fast and reliable simultaneous eeg-fmri analysis of epilepsy with automatic spike detection. Clin Neurophysiol.

[5] Seneviratne U, Karoly P, Freestone DR, Cook MJ, Boston RC (2019). Methods for the detection of seizure bursts in epilepsy. Front Neurol..

[6] Zhang Y, Guo Y, Yang P, Chen W, Lo B (2019). Epilepsy seizure prediction on eeg using common spatial pattern and convolutional neural network. IEEE J Biomed Health Inform.

[7] van Straaten EC, Stam CJ (2013). Structure out of chaos: functional brain network analysis with eeg, meg, and functional mri. Eur Neuropsychopharmacol.

[8] Ozcan AR, Erturk S (2019). Seizure prediction in scalp eeg using 3d convolutional neural networks with an image-based approach. IEEE Trans Neural Syst Rehabil Eng.

[9] Schindler KA, Bialonski S, Horstmann MT, Elger CE, Lehnertz K (2008). Evolving functional network properties and synchronizability during human epileptic seizures. Chaos.

[10] Mei T, Wei X, Chen Z, Tian X, Dong N, Li D, Zhou Y (2019). Epileptic foci localization based on mapping the synchronization of dynamic brain network. BMC Med Inform Decis Mak.

[11] Chamseddine A, Sawan M. Deep learning based method for output regularization of the seizure prediction classifier. In: 2018 IEEE Life Sciences Conference (LSC), pp 118–121. IEEE

[12] Talathi,SS. Deep recurrent neural networks for seizure detection and early seizure detection systems. 2017. arXiv preprint arXiv:1706.03283

[13] Wang Y, Jiang L, Yang M, Li L, Long M, Feifei L. Eidetic 3d lstm: A model for video prediction and beyond. In: International conference on learning representations

[14] van Mierlo P, Papadopoulou M, Carrette E, Boon P, Vandenberghe S, Vonck K, Marinazzo D (2014). Functional brain connectivity from eeg in epilepsy: seizure prediction and epileptogenic focus localization. Prog Neurobiol.

[15] Zhang Z, Chen Z, Zhou Y, Du S, Zhang Y, Mei T, Tian X (2014). Construction of rules for seizure prediction based on approximate entropy. Clin Neurophysiol.

[16] Strogatz SH. Nonlinear dynamics and chaos with student solutions manual: with applications to physics, biology, chemistry, and engineering. 2018.

[17] Ravindra VM, Sweney MT, Bollo RJ (2017). Recent developments in the surgical management of paediatric epilepsy. Arch Dis Child.

[18] Bandyopadhyay S, Koubeissi MZ, Azar NJ. Physiologic basis of eeg and epilepsy. 2017.

[19] Caballero-Gaudes C, Van de Ville D, Grouiller F, Thornton R, Lemieux L, Seeck M, Lazeyras F, Vulliemoz S (2013). Mapping interictal epileptic discharges using mutual information between concurrent eeg and fmri. Neuroimage.

[20] Sharmila A, Geethanjali P (2016). Detection of epileptic seizure from electroencephalogram signals based on feature ranking and best feature subset using mutual information estimation. J Med Imaging Health Inform.

[21] Li FL, Liang Y, Zhang LY, Yi CL, Liao YY, Jiang YL, Si YJ, Zhang YS, Yao DZ, Yu L, Xu P (2019). Transition of brain networks from an interictal to a preictal state preceding a seizure revealed by scalp eeg network analysis. Cogn Neurodyn.

[22] Zhang Z, Zhou Y, Mei T, Chen ZY, Zhou Y, Du SH, Tian XH. Localization of epileptic foci based on scalp eeg and approximate entropy. In: Proceedings of the 2013 6th international conference on biomedical engineering and informatics (Bmei 2013), Vols 1 and 2, 240–244. 2013.

[23] Faust O, Acharya UR, Adeli H, Adeli A (2015). Wavelet-based eeg processing for computer-aided seizure detection and epilepsy diagnosis. Seizure.

[24] Juarez-Martinez EL, Nissen IA, Idema S, Velis DN, Hillebrand A, Stam CJ, van Straaten ECW (2018). Virtual localization of the seizure onset zone: Using non-invasive meg virtual electrodes at stereo-eeg electrode locations in refractory epilepsy patients. Neuroimage-Clin.

[25] Repovs G, Csernansky JG, Barch DM (2011). Brain network connectivity in individuals with schizophrenia and their siblings. Biol Psychiatry.

[26] Viglione SS, Walsh GO (1975). Proceedings: epileptic seizure prediction. Electroencephalogr Clin Neurophysiol.

[27] Smith EH, Schevon CA (2016). Toward a mechanistic understanding of epileptic networks. Curr Neurol Neurosci Rep.

[28] Su L, An J, Ma Q, Qiu S, Hu D (2015). In uence of resting-state network on lateralization of functional connectivity in mesial temporal lobe epilepsy. Am J Neuroradiol.

[29] Goodfellow M, Rummel C, Abela E, Richardson MP, Schindler K, Terry JR (2016). Estimation of brain network ictogenicity predicts outcome from epilepsy surgery. Sci Rep.

[30] Gomez-Pilar J, Poza J, Gomez C, Northoff G, Lubeiro A, Cea-Canas BB, Molina V, Hornero R (2018). Altered predictive capability of the brain network eeg model in schizophrenia during cognition. Schizophr Res.

[31] Supriya S, Siuly S, Wang H, Cao JL, Zhang YC (2016). Weighted visibility graph with complex network features in the detection of epilepsy. Ieee Access.

[32] Adebimpe A, Aarabi A, Bourel-Ponchel E, Mahmoudzadeh M, Wallois F (2016). Eeg resting state functional connectivity analysis in children with benign epilepsy with centrotemporal spikes. Front Neurosci.

[33] Allen EA, Damaraju E, Eichele T, Wu L, Calhoun VD (2018). Eeg signatures of dynamic functional network connectivity states. Brain Topogr.

[34] Martis RJ, Acharya UR, Tan JH, Petznick A, Tong L, Chua CK, Ng EY (2013). Application of intrinsic time-scale decomposition (itd) to eeg signals for automated seizure prediction. Int J Neural Syst.

[35] Osorio I, Harrison MA, Lai YC, Frei MG (2001). Observations on the application of the correlation dimension and correlation integral to the prediction of seizures. J Clin Neurophysiol.

